# Exploring the Molecular Mechanism of Cinnamaldehyde Intervening in Ochratoxin A-Induced Type 2 Diabetes Mellitus and Non-Alcoholic Fatty Liver Disease Comorbidity: An Integrated Approach Based on Network Pharmacology, Network Toxicology and Molecular Docking

**DOI:** 10.3390/ph19081283

**Published:** 2026-08-13

**Authors:** Mingli Shen, Qingping Shi, Shuang Gao, Beiyan Chen, Jieru Han

**Affiliations:** College of Basic Medicine, Heilongjiang University of Chinese Medicine, Harbin 150040, China; 18309474893@163.com (M.S.); sqp17326705862@163.com (Q.S.); gaoshuangacc@163.com (S.G.); chenbeiyanabc@163.com (B.C.)

**Keywords:** cinnamaldehyde, ochratoxin A, type 2 diabetes mellitus and non-alcoholic fatty liver disease, comorbidity analysis, network toxicology, network pharmacology, molecular docking

## Abstract

**Background/Objective:** Cinnamaldehyde (CA) is a naturally occurring bioactive compound derived from the leaves, bark, roots, and flowers of the Chinese medicinal plant *Cinnamomum cassia*. It exhibits a broad spectrum of pharmacological properties, encompassing antioxidant, antibacterial, anti-diabetic, antifungal, and anticancer activities. Notably, it has shown potential therapeutic benefits in the management of type 2 diabetes mellitus (T2DM) and non-alcoholic fatty liver disease (NAFLD). Ochratoxin A (OTA), a common contaminant found in foods such as cereals, coffee, and raisins, is also present in traditional Chinese medicinal materials, including *Astragalus* and *liquorice*. T2DM and NAFLD share intertwined pathophysiological pathways, including insulin resistance, dyslipidaemia, chronic low-grade inflammation and oxidative stress, with insulin resistance serving as the common pathological hub for both conditions. Consequently, they frequently co-occur and exacerbate each other. OTA exerts dual-targeted toxicity to the pancreas and liver, which may synergistically drive the development of the comorbidity of T2DM and NAFLD. These two processes are mutually causal and together constitute the pathological basis of metabolic comorbidity. **Methods:** Network toxicology employs toxicological data, gene expression, and protein–protein interaction (PPI) networks to predict the targets of toxins, while network pharmacology, based on systems biology principles, reveals how drugs exert regulatory effects through multiple targets and pathways. In this study, we employed an integrated network toxicology and network pharmacology approach to jointly decipher the potential mechanisms by which CA intervenes in OTA-induced comorbid T2DM-NAFLD. First, a network toxicology approach was employed to preliminarily screen for core toxicological targets responsible for OTA’s pathogenicity. Subsequently, network pharmacology was used to identify potential targets of CA-mediated intervention in the disease. Finally, the common overlap among the CA intervention targets, OTA toxicity targets, and disease targets was defined as the final set of potential targets for CA-mediated intervention in OTA-induced T2DM-NAFLD comorbidity. A PPI network was constructed using the STRING database, and topological analysis was performed with Cytoscape. Core targets were selected using the median values of six parameters—betweenness centrality, closeness centrality, degree centrality, eigenvector centrality, LAC (local average connectivity) score, and network centrality—as cut-off thresholds, and the top 10 key genes were further identified using the cytoHubba plugin. Gene Ontology (GO) functional enrichment and Kyoto Encyclopedia of Genes and Genomes (KEGG) pathway enrichment analyses were conducted via the DAVID database, and the results were visualized on the CNSknowall platform. Lastly, molecular docking of the core targets was performed using the CB-DOCK2 platform to validate binding affinity. **Results:** Based on an integrated analysis of network toxicology, network pharmacology, and molecular docking, 10 key targets were systematically identified. These may serve as potential mediators of cinnamaldehyde in the treatment of OTA-induced T2DM-NAFLD comorbidity. Among these, six targets—albumin (*ALB*), glyceraldehyde-3-phosphate dehydrogenase (*GAPDH*), interleukin-6 (*IL-6*), tumor necrosis factor (*TNF*), actin beta (*ACTB*), and estrogen receptor 1 (*ESR1*)—possess crystal structures amenable to molecular docking. KEGG enrichment analysis revealed that CA and OTA jointly participate in key pathological processes such as the cancer pathway, the lipid and atherosclerosis pathway, the advanced glycation end-products–receptor for advanced glycation end-products (*AGE-RAGE*) signaling pathway, the phosphatidylinositol 3-kinase–protein kinase B (*PI3K-Akt*) signaling pathway, the TNF signaling pathway, and the interleukin-17 (*IL-17*) signaling pathway. OTA exacerbates inflammatory responses, impairs insulin signaling, promotes hepatic steatosis, and disrupts systemic metabolic homeostasis, ultimately contributing to T2DM-NAFLD comorbidity. Conversely, cinnamaldehyde counteracts these pathological processes through multiple mechanisms, including antioxidant and anti-inflammatory effects as well as regulation of glucose and lipid metabolism, thereby restoring metabolic homeostasis. **Conclusions:** This study has preliminarily identified the toxicological targets of OTA and the potential intervention targets of CA, offering new avenues for preventing and intervening in OTA-induced metabolic toxicity. Furthermore, it provides a theoretical basis for CA as a potential multi-target therapeutic agent and presents novel insights worthy of further investigation into the prevention of T2DM-NAFLD comorbidity.

## 1. Introduction

Type 2 diabetes mellitus (T2DM) and non-alcoholic fatty liver disease (NAFLD) frequently co-occur, constituting a complex metabolic syndrome. The pathogenesis of this condition is intricate and closely associated with exposure to environmental toxins, which contributes to the continuous rise in its global prevalence [1]. Studies indicate that the global prevalence of NAFLD, also referred to as metabolic dysfunction-associated steatotic liver disease (MASLD), among individuals with type 2 diabetes is 65.33% (95%CI, 62.35–68.18%). This prevalence increased from 55.86% (42.38–68.53%) in 1990–2004 to 68.81% (63.41–73.74%) in 2016–2021 [2], and has now become a major global public health challenge. The disease spectrum of NAFLD ranges from simple hepatic steatosis to non-alcoholic steatohepatitis, and may advance to fibrosis, cirrhosis, and even hepatocellular carcinoma [3]. The prevalence of NAFLD is extremely high among patients with T2DM; epidemiological data indicate that approximately 60–80% of T2DM patients also have NAFLD [4]. A study using transient elastography found that the prevalence of NAFLD among T2DM patients was as high as 72.4%, with 21.0% of these patients exhibiting advanced liver fibrosis [5]. The pathophysiological mechanism underlying T2DM–NAFLD comorbidity is not attributable to a single factor, but rather forms a vicious cycle: NAFLD promotes the onset and progression of T2DM by exacerbating hepatic insulin resistance and lipotoxicity [6]; conversely, T2DM, via hyperglycemia, hyperinsulinemia and dyslipidemia, accelerates hepatic steatosis, inflammatory infiltration, and even fibrosis in NAFLD [7]. Furthermore, chronic inflammation is considered a key link between T2DM and NAFLD; the release of pro-inflammatory cytokines exacerbates hepatic inflammatory damage and further worsens systemic insulin resistance. This comorbidity not only exacerbates patients’ metabolic disorders but also significantly increases the risk of cardiovascular disease and end-stage liver disease [8]. Therefore, in-depth research into the pathogenesis of T2DM-NAFLD comorbidity and the identification of effective intervention targets is of great clinical significance.

Ochratoxin A (OTA) is a secondary metabolite produced by *Aspergillus* and *Penicillium fungi*. This substance is widely found in a variety of products, including traditional Chinese medicinal materials, cereals, coffee, and raisins, posing a serious threat to human and animal health [9]. Existing research has found that ochratoxin contamination in traditional Chinese medicinal materials and prepared herbal slices is relatively severe [10]. For example, among 176 samples of *liquorice*, the OTA contamination rate reached as high as 66.5%, with some samples exceeding the European Union limit [11]. The International Agency for Research on Cancer (IARC) has classified it as a Group 2B carcinogen [12]. The primary mechanisms of OTA toxicity are complex and include the induction of oxidative stress, inhibition of protein synthesis, disruption of cellular signal transduction, and triggering of apoptosis [13]. It is worth noting that OTA has a relatively long half-life in the body, approximately 35 days, and tends to accumulate in tissues, thereby exacerbating its long-term toxicity [14]. Its toxicological effects are wide-ranging, involving nephrotoxicity, hepatotoxicity, neurotoxicity, immunotoxicity, and potential carcinogenicity [9,15]. In recent years, a growing body of evidence suggests that OTA exposure may be associated with metabolic disorders; for example, it can affect lipid metabolism and induce oxidative stress, endoplasmic reticulum stress, mitochondrial dysfunction, and chronic inflammatory responses [16,17]. Several studies have confirmed that OTA can directly promote the onset and progression of T2DM-NAFLD comorbidity by inducing insulin resistance and hepatic steatosis [18,19].

Cinnamaldehyde (CA) is a natural bioactive compound extracted from the Chinese medicinal herb *Cinnamomum cassia*. CA has been demonstrated to possess a variety of biological activities, including anti-inflammatory and antioxidant effects, as well as improving insulin resistance and regulating lipid metabolism. In recent years, it has attracted widespread attention in the field of metabolic disease treatment [20,21]. Studies have shown that CA can attenuate oxidative stress by activating the nuclear factor erythroid 2-related factor 2 (Nrf2)/antioxidant response element (ARE) signaling pathway, thereby enhancing the activity of antioxidant enzymes [22]. Furthermore, CA exerts anti-inflammatory effects by inhibiting the nuclear factor kappa-light-chain-enhancer of activated B cells (NF-κB) pathway and reducing the expression of pro-inflammatory cytokines such as tumor necrosis factor-α (TNF-α), interleukin-6 (IL-6), and interleukin-1β (IL-1β) [23]; these effects are closely associated with the improvement in NAFLD. Additionally, CA can improve insulin signaling by enhancing the phosphorylation of insulin receptor substrate-1 (IRS-1), thereby lowering blood glucose levels and showing potential therapeutic value for T2DM [24]. Although CA has demonstrated favorable intervention effects in single metabolic disorders, its therapeutic mechanism in OTA-induced T2DM-NAFLD comorbidity remains unclear. This uncertainty severely hampers its precise clinical application and drug development. Therefore, investigating the molecular basis and mechanisms of CA in OTA-induced T2DM-NAFLD comorbidity is of great value in guiding novel clinical treatment strategies.

With the rapid development of bioinformatics and big data analysis, the integration of informatics technologies plays a significant role in elucidating the mechanisms of Traditional Chinese Medicine (TCM) treatment and demonstrates vast potential in analyzing comorbidities. Against this backdrop, network pharmacology has become a key method for analyzing the inherently complex systems of TCM and is often combined with molecular approaches; network toxicology, meanwhile, analyzes the disruptive effects of environmental toxins on biological networks from the perspective of systems toxicology. This study combines network toxicology and network pharmacology to investigate the potential mechanisms through which CA exerts multi-target effects against OTA-induced T2DM-NAFLD comorbidity. Consequently, this study systematically identifies key targets and signaling pathways, providing a scientific basis for the development of CA-based drugs and offering new insights into the prevention and treatment of T2DM-NAFLD comorbidity.

## 2. Results

### 2.1. Gene Landscape of Type 2 Diabetes Mellitus and Non-Alcoholic Fatty Liver Disease Co-Expression

**Figure 1:**
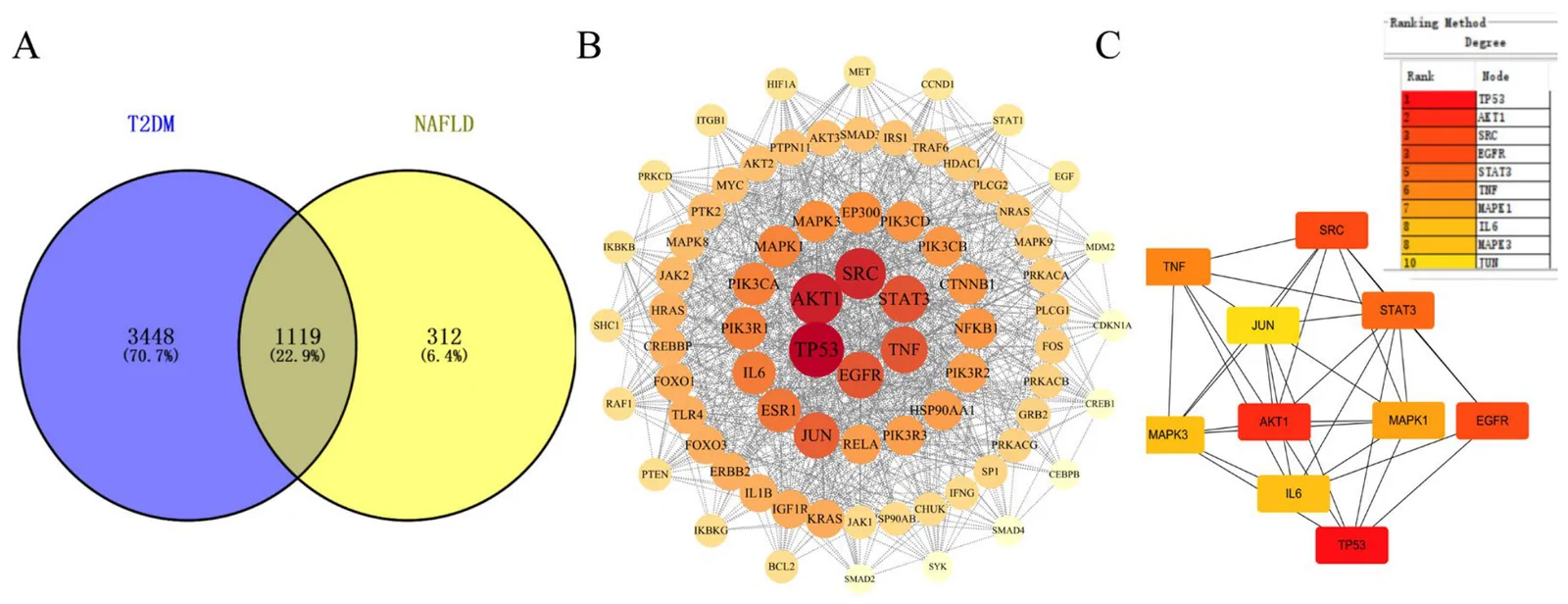
Identification of targets associated with T2DM and NAFLD. (**A**) A Venn diagram of targets associated with T2DM and NAFLD generated using Venny 2.1. Blue and yellow circles represent T2DM and NAFLD-associated targets, respectively; the overlapping region indicates the intersection of the two sets. (**B**,**C**) A PPI network of the core targets screened using the CytoNCA plugin in Cytoscape 3.10.1, and the top 10 hub genes identified by the Degree algorithm. Nodes represent proteins, while edges represent interactions between proteins. Node color and size are mapped to degree values: a gradient from yellow to red and increasing node size indicate higher centrality within the network.

The Comparative Toxicogenomics Database (CTD) is a comprehensive resource designed to advance research in toxicology, genomics, and environmental health. It integrates information on chemical–gene, chemical–phenotype, and chemical–disease interactions from diverse sources and provides interactive query and network analysis functions. The CTD can be used to investigate mechanistic hypotheses regarding the effects of environmental toxicants on disease and supports the development of adverse outcome pathways (AOPs). Disease keywords were searched in the CTD (https://ctdbase.org/; accessed on 6 June 2026) and the GeneCards database (https://www.genecards.org/; accessed on 6 June 2026). The identified genes were imported into Venny 2.1 (https://bioinfogp.cnb.csic.es/tools/venny/; accessed on 7 June 2026); the resulting set of common genes served as potential targets associated with both T2DM and NAFLD. A total of 1119 genes were found to be associated with both diseases (Figure 1A). Subsequently, a PPI network was constructed for the intersecting targets using the STRING database (version 12.0; https://string-db.org/; accessed on 7 June 2026), with the species set to homo sapiens and a minimum required interaction score of ≥0.4. The network was imported into Cytoscape 3.10.1 (https://cytoscape.org/; accessed on 9 October 2025), and the following node parameters were calculated: degree, closeness centrality, betweenness centrality, eigenvector centrality, network structure, and LAC (local average connectivity) score. To ensure an objective and unbiased selection, the values of these six parameters were determined for all nodes in the PPI network, and the median of each parameter was uniformly adopted as the cut-off threshold. Core targets were preliminarily selected according to the following criteria: betweenness ≥ 128.54, closeness ≥ 0.43, degree ≥ 19, eigenvector ≥ 0.04, LAC ≥ 7.68, and network structure ≥ 8.88. After this screening, a total of 76 targets were identified, and a subnetwork was constructed using these 76 targets (Figure 1B). Finally, core genes were identified by applying the Degree algorithm in the CytoHubba plugin of Cytoscape (Figure 1C).

**Figure 2:**
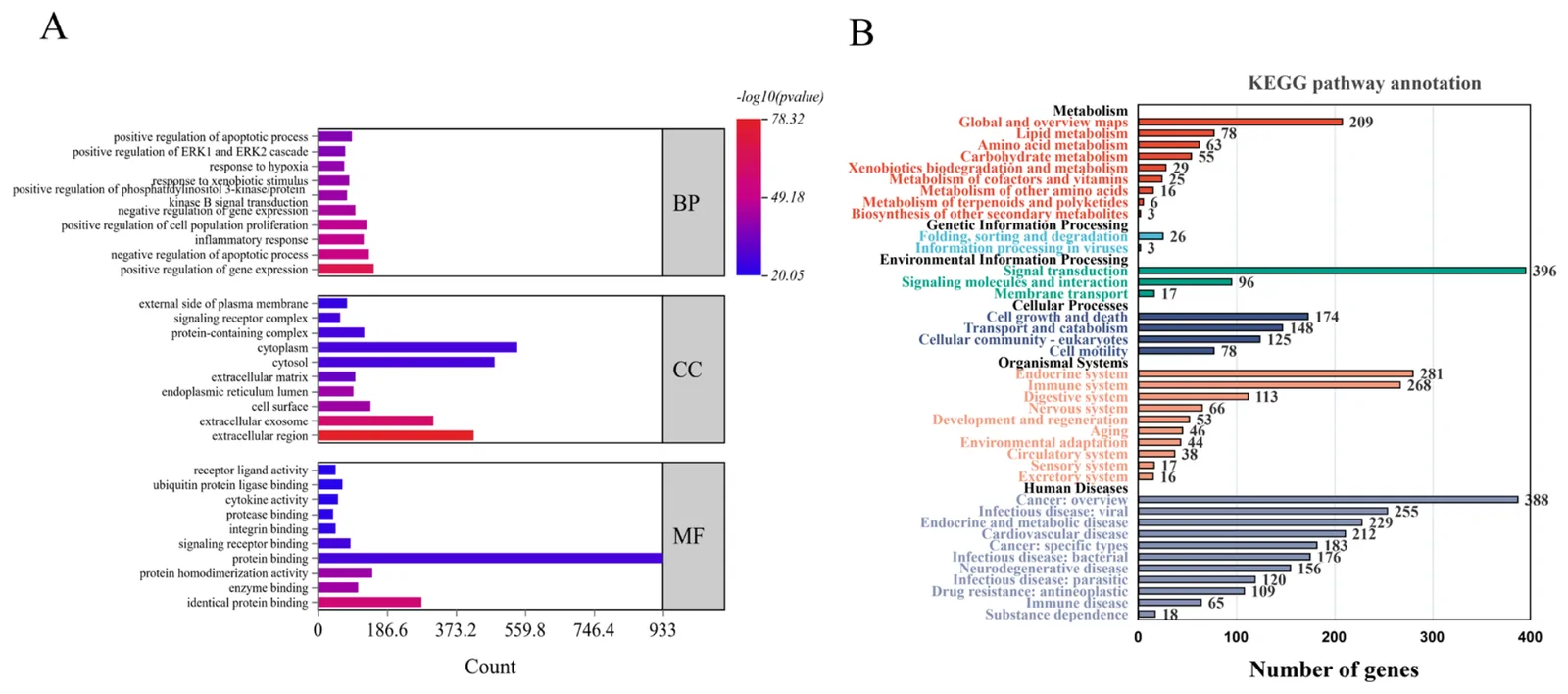
Enrichment analysis of targets associated with T2DM and NAFLD (**A**) GO functional enrichment analysis generated using the CNSknowall online platform. The x-axis (Count) represents the number of core target genes enriched in a given GO term, and the y-axis represents the specific GO functional annotation terms. The color bar on the right shows −log10 (*p*-value), with colors ranging from blue (lower values, lower significance) to red (higher values, greater significance). A redder color indicates a more statistically significant enrichment result for that biological process, i.e., a stronger association between that process and the core target genes. (**B**) Enrichment analysis of the top 20 KEGG pathways, sorted in descending order by GeneRatio and generated using the CNSknowall online platform. The x-axis (Number of genes) represents the number of core target genes contained within each KEGG pathway, and the y-axis represents the specific KEGG pathway names along with their corresponding broad biological categories. Color coding of the categories: Metabolism, reddish-brown; Genetic Information Processing, pale blue; Environmental Information Processing, dark green; Cellular Processes, dark blue; Organismal Systems, orange; Human Diseases, blue-purple. The number to the right of each bar indicates the specific number of enriched genes in that pathway. The figure reflects the ranking by gene count; the longer the bar, the more core target genes the pathway involves.

GO functional enrichment analysis (Figure 2A) revealed that these genes are primarily involved in multiple biological pathways, are distributed across various subcellular functional domains, and mediate diverse protein–protein interaction. They link key pathological mechanisms—such as insulin resistance, chronic low-grade inflammation, hepatocyte damage and tissue fibrosis—in the comorbidity processes of these two metabolic diseases. KEGG enrichment analysis (Figure 2B) revealed that the ‘Environmental Information Processing’ pathway exhibited the highest level of enrichment; within this major category, ‘Signal transduction’ was the sub-pathway with the highest number of annotated genes, suggesting that abnormalities in cellular signal transduction represent the core molecular mechanism mediating the onset and progression of the comorbidity of type 2 diabetes and non-alcoholic fatty liver disease. This was followed by ‘Human Diseases’, ‘Organismal Systems’ and ‘Metabolism’, whilst the ‘Genetic Information Processing’ pathway had the fewest annotated genes. At the sub-pathway level, significant gene enrichment was also observed in ‘General Oncology’ and ‘Endocrine and Metabolic Diseases’ under the ‘Human Diseases’ category; in the ‘Organismal Systems’, ‘Endocrine System’ and in ‘Metabolism’ categories, the ‘Lipid’ and ‘Carbohydrate Metabolism’ pathways were significantly enriched. Collectively, these findings reflect that disturbances in glucose and lipid metabolism, endocrine and immune imbalances, and multi-system pathologies are pathological features shared by both disease types.

### 2.2. Toxicological Study of Ochratoxin A-Induced Type 2 Diabetes Mellitus and Non-Alcoholic Fatty Liver Disease Complications Based on Network Toxicology

#### 2.2.1. Prediction of OTA and Targets Associated with This Complication

**Figure 3:**
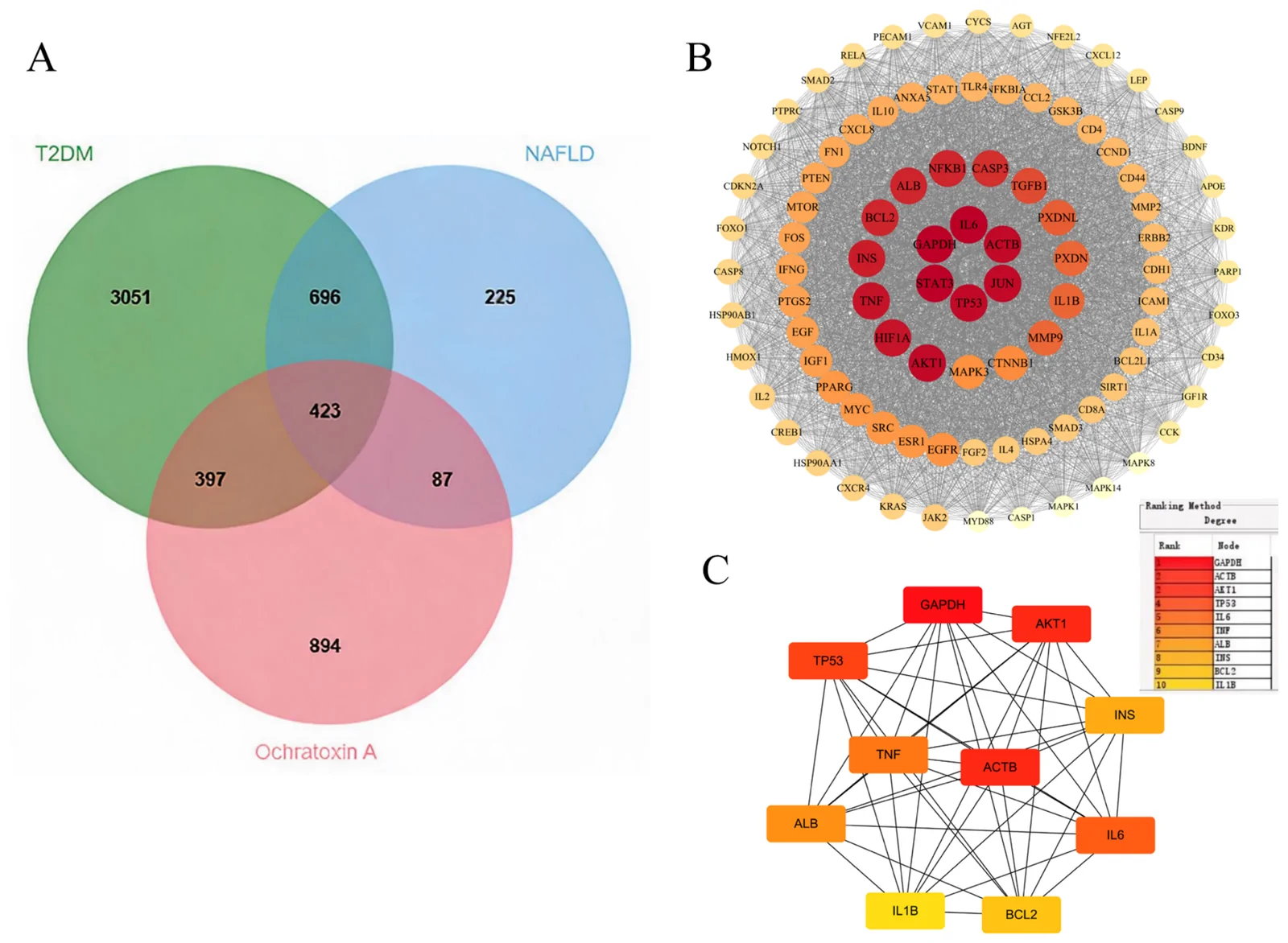
Analysis of the association between OTA and T2DM-NAFLD comorbidity. (**A**) A Venn diagram of targets associated with OTA and T2DM-NAFLD comorbidity, generated using the Venny 2.1 online tool. Green, blue, and pink circles represent T2DM-associated, NAFLD-associated, and OTA-associated targets, respectively. (**B**,**C**) A PPI network of the core targets screened using the CytoNCA plugin in Cytoscape 3.10.1, and the top 10 hub genes identified by the Degree algorithm. Nodes represent proteins, while edges represent interactions between proteins. Node color and size are mapped to degree values: a gradient from yellow to red and increasing node size indicate higher centrality within the network.

We conducted comprehensive target prediction using the CTD and the GeneCards database, identifying 1317 potential targets for OTA and 3972 for T2DM-NAFLD. To identify the core regulatory nodes linking OTA and T2DM-NAFLD pathogenesis, we analyzed the intersection of the two target sets, which yielded 423 overlapping targets (Figure 3A). Given that these results are based solely on database-driven predictions, we tentatively regard these 423 targets as potential targets for OTA-induced T2DM-NAFLD complications.

#### 2.2.2. Protein–Protein Interaction Network Analysis of Ochratoxin A-Targeted Molecules in the Comorbid Conditions

The overlapping targets were imported into the STRING database to generate a PPI network. The resulting interaction data were imported into Cytoscape 3.10.1, and key targets were screened using the following criteria: betweenness ≥ 44.32, closeness ≥ 0.71, degree ≥ 118, eigenvector ≥ 0.07, LAC ≥ 91.83, and network ≥ 96.84. After this screening, 93 core targets were identified and visualized in the PPI network (Figure 3B). These targets were further analyzed using the Degree algorithm in the CytoHubba plugin, and the top 10 core targets—*GAPDH*, *ACTB*, *AKT1*, *TP53*, *IL6*, *TNF*, *ALB*, *INS*, *BCL2*, and *IL1B*—were identified as potential targets for OTA-induced T2DM-NAFLD comorbidity (Figure 3C).

#### 2.2.3. Gene Ontology Analysis and Kyoto Encyclopedia of Genes and Genomes Pathway Enrichment Analysis of Ochratoxin A-Targeted Molecules in the Comorbid Conditions

**Figure 4:**
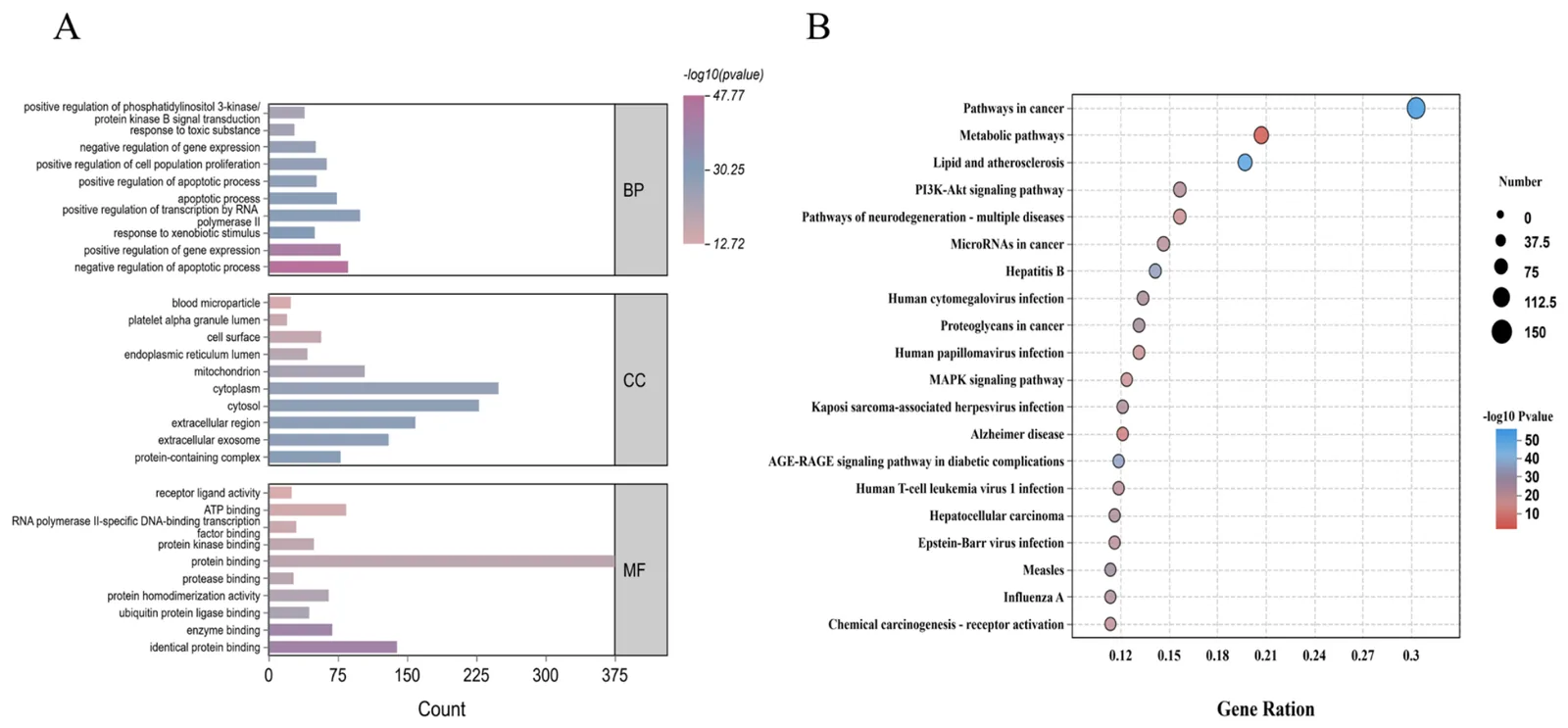
Enrichment analysis of OTA and T2DM-NAFLD comorbidity. (**A**) GO functional enrichment analysis generated using the CNSknowall online platform. The x-axis represents count, and the y-axis shows the GO functional terms, categorized into three major classes: BP, CC, and MF. The color gradient reflects the significance level, i.e., −log10(*p*-value), with a transition from purple to pink indicating increasing significance (higher values correspond to more significant enrichment). (**B**) KEGG pathway enrichment analysis generated using the CNSknowall online platform. The x-axis represents the gene ratio (the proportion of enriched genes relative to the total input genes), and the y-axis shows the KEGG pathway names. The dot size indicates the number of enriched genes; larger dots represent more enriched genes. The dot color represents the significance level, i.e., −log10(*p*-value), with a gradient from blue to red indicating a gradual increase in significance.

To elucidate the toxicological mechanisms by which OTA induces T2DM-NAFLD comorbidity, we performed GO and KEGG enrichment analyses on the 93 core targets using the DAVID database [25], with homo sapiens selected as the reference species. A total of 203 KEGG pathways and 1835 GO terms were identified. For GO enrichment, we selected the top 10 terms with the highest gene proportion in each of the three categories—BP, CC, and MF—and generated a GO enrichment plot (Figure 4A). For KEGG enrichment, the top 20 pathways sorted by GeneRatio were visualized as a bubble chart (Figure 4B).

The results of the GO enrichment analysis show that, at the biological process level, the target genes are predominantly involved in PI3K/AKT insulin signaling, regulation of the apoptosis–proliferation balance, RNA polymerase II-dependent transcriptional regulation, and the response to exogenous toxins, with dysregulation of the apoptotic balance emerging as the most significant core biological process. At the cellular component level, the relevant proteins are primarily localized in key subcellular structures involved in glycolipid metabolism and cell-to-cell communication, such as the cytoplasm, mitochondria, endoplasmic reticulum, and extracellular exosomes, suggesting that OTA specifically disrupts energy metabolism, lipid synthesis, and interorgan vesicular communication in hepatocytes. At the molecular function level, protein binding was the predominant function, alongside widespread enrichment of terms such as kinase binding, enzyme binding, transcription factor DNA binding, and receptor–ligand interactions. This indicates that OTA disrupts insulin signaling and the hepatic lipid metabolism network via multiple targets by interfering with protein interactions, post-translational modifications, and gene transcription, ultimately inducing the comorbidity of T2DM and NAFLD.

The results of the KEGG enrichment analysis show that the core enriched pathways are pathways in cancer, metabolic pathways, lipid and atherosclerosis, the AGE-RAGE signaling pathway associated with diabetic complications, and the PI3K-Akt insulin signaling pathway. These pathways directly regulate the body’s glucose and lipid metabolic homeostasis, insulin signaling, hepatic lipid accumulation, and chronic inflammatory damage associated with diabetes, which aligns well with the pathological characteristics of T2DM-NAFLD comorbidity. At the inflammatory level, the MAPK signaling pathway mediates OTA-induced chronic inflammatory stress in liver tissue; neurodegenerative-related pathways suggest that OTA may exacerbate metabolic disorders through interactions via the gut–liver–brain axis, while viral infection, hepatocellular carcinoma, and tumor-related pathways reveal that long-term exposure to OTA can simultaneously induce liver damage and increase the risk of malignant liver lesions through persistent metabolic toxicity and chronic inflammation. The overall pathway enrichment results confirm that OTA disrupts glucose and lipid metabolism, insulin signaling, and the oxidative–inflammatory balance through the synergistic action of multiple pathways, driving insulin resistance, hepatic steatosis, and chronic liver damage multidimensionally, ultimately mediating the onset and progression of T2DM-NAFLD comorbidity.

### 2.3. Network Pharmacology-Based Investigation of Mechanisms of Action of Cinnamaldehyde in Treating Type 2 Diabetes Mellitus and Non-Alcoholic Fatty Liver Disease Comorbidity

#### 2.3.1. Target Prediction

**Figure 5:**
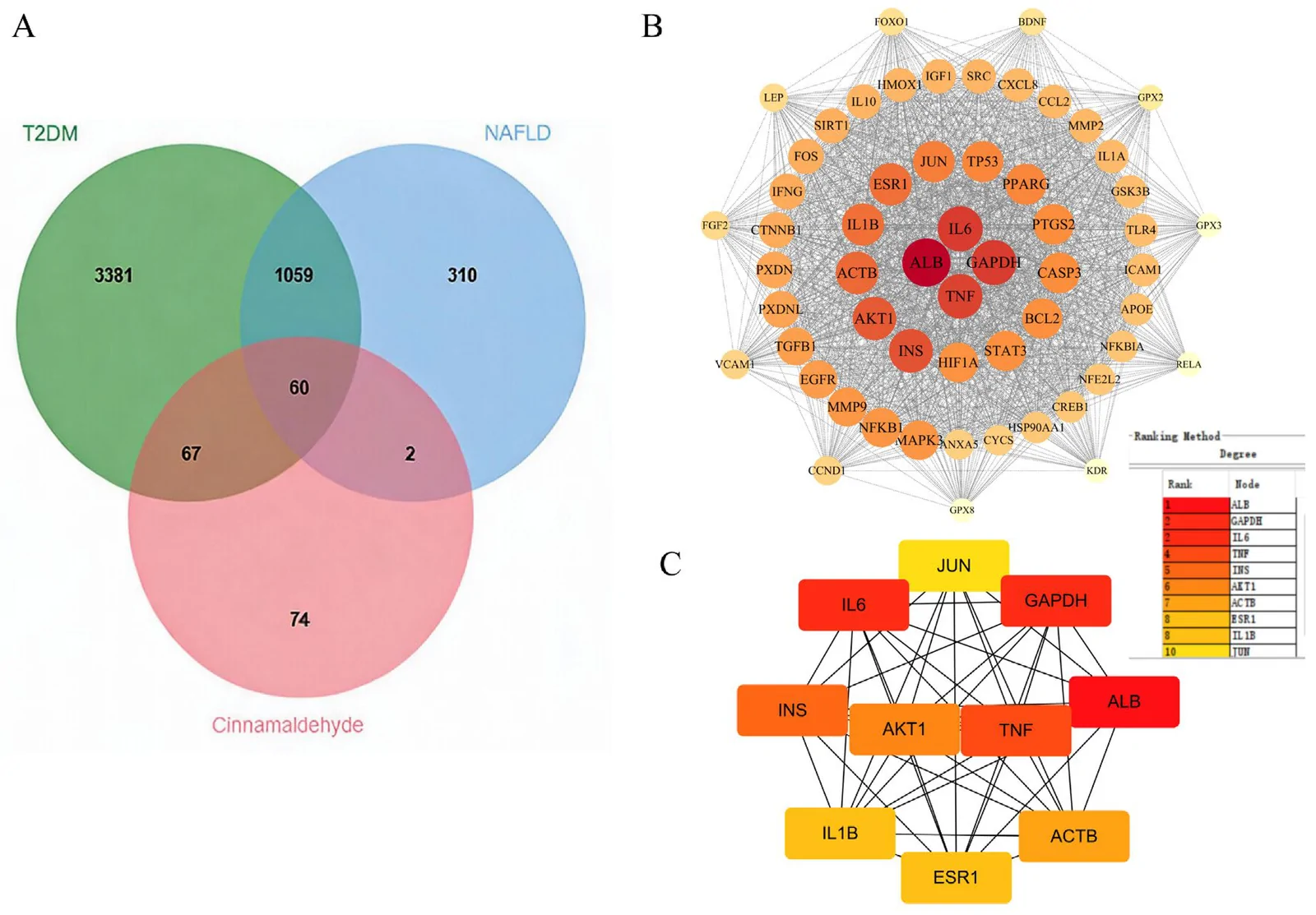
Analysis of the association between CA and T2DM-NAFLD comorbidity. (**A**) A Venn diagram of CA, T2DM, and NAFLD-associated target overlaps generated using Venny 2.1. Green, blue, and pink circles represent T2DM, NAFLD, and CA-associated targets, respectively. (**B**,**C**) A PPI network of the core targets screened using the CytoNCA plugin in Cytoscape 3.10.1, and the top 10 hub genes identified by the Degree algorithm. Nodes represent proteins, while edges represent interactions between proteins. Node color and size are mapped to degree values: a yellow-to-red gradient and increasing node size indicate higher centrality within the network.

Comprehensive target prediction for CA was performed using the CTD and the GeneCards database, yielding 203 potential targets for CA and 3972 for T2DM-NAFLD. Intersection analysis of these two target sets identified 60 core targets (Figure 5A). Given that these results are based solely on database-driven predictions, these 60 targets were provisionally regarded as potential therapeutic targets for the treatment of T2DM-NAFLD comorbidity with CA.

#### 2.3.2. Protein–Protein Interaction Network Analysis of Cinnamaldehyde in the Comorbid Conditions

The 60 overlapping targets were imported into the STRING database to generate a PPI network. The resulting network was then imported into Cytoscape 3.10.1, and key targets were screened using the following criteria: betweenness ≥ 46.99, closeness ≥ 0.65, degree ≥ 75.5, eigenvector ≥ 0.08, LAC ≥ 59.90, and network ≥ 64.80. After this screening, 57 targets were identified and visualized in the PPI network (Figure 5B). Using the Degree algorithm in the CytoHubba plugin, the top 10 core targets—ALB, GAPDH, IL6, TNF, INS, AKT1, ACTB, ESR1, IL1B, and JUN—were identified as potential targets for cinnamaldehyde therapy against T2DM-NAFLD comorbidity (Figure 5C).

#### 2.3.3. Gene Ontology Analysis and Kyoto Encyclopedia of Genes and Genomes Pathway Enrichment Analyses of Target Molecules Involved in Cinnamaldehyde Treatment of Comorbid Conditions

**Figure 6:**
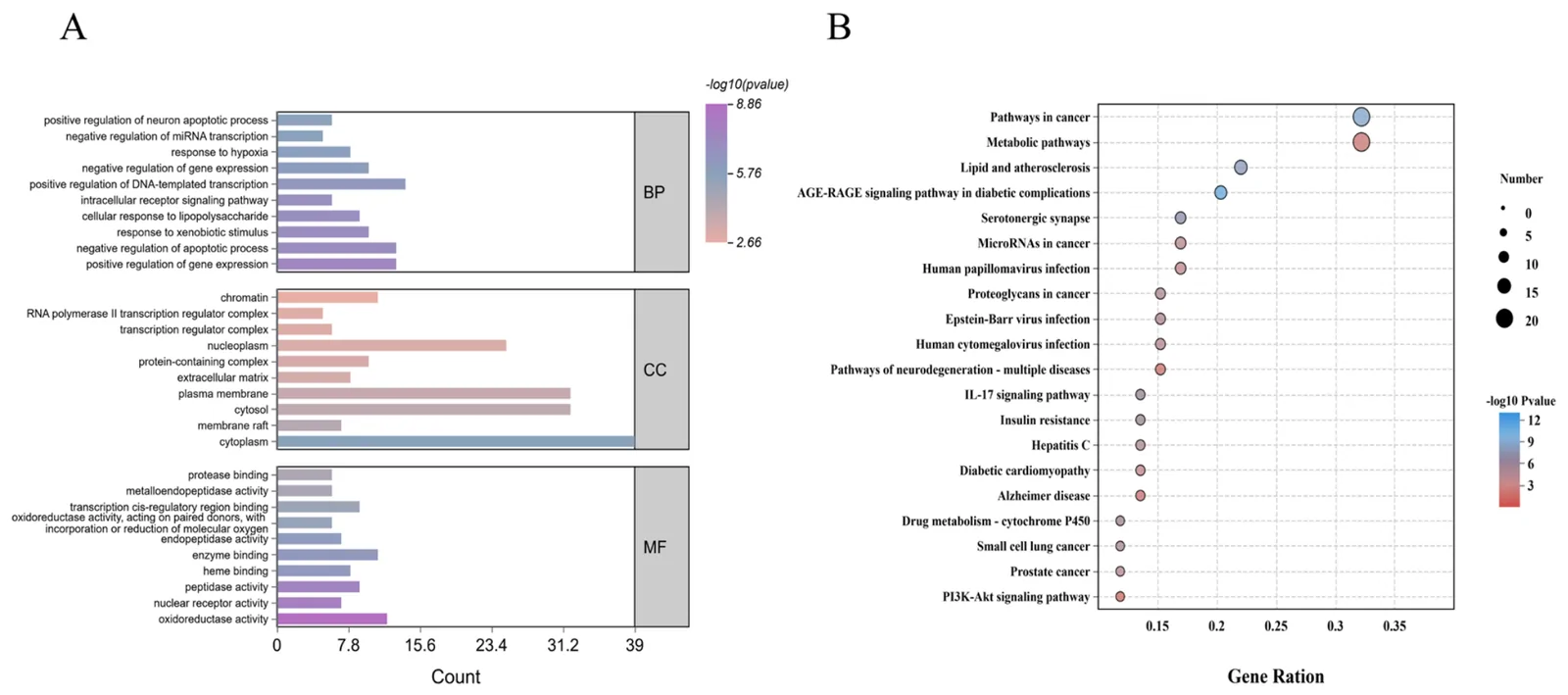
Enrichment analysis of CA targets in T2DM-NAFLD comorbidity. (**A**) GO functional enrichment analysis generated using the CNSknowall online platform. The x-axis represents count, and the y-axis shows the GO functional terms, categorized into three major classes: BP, CC, and MF. The color gradient indicates the significance level, i.e., −log10(*p*-value), with a transition from purple to pink representing increasing significance (higher values correspond to more significant enrichment). (**B**) KEGG pathway enrichment analysis generated using the CNSknowall online platform. The x-axis represents the gene ratio (the proportion of enriched genes relative to the total input genes), and the y-axis shows the KEGG pathway names. The dot size represents the number of enriched genes, with larger dots indicating more enriched genes. The dot color indicates the significance level, i.e., −log10(*p*-value), with a gradient from blue to red representing a gradual increase in significance.

The results of the GO enrichment analysis (Figure 6A) show that biological functions exhibit multi-level regulatory characteristics: at the biological process level, the core processes are the regulation of apoptosis balance, transcriptional network remodeling, and metabolic stress response; at the cellular component level, targets are primarily distributed in the cytoplasm/plasma membrane signaling domains, nuclear transcription complexes, and the extracellular matrix; at the molecular function level, the targets primarily exert effects through redox enzyme activation, proteolytic hydrolysis, binding to transcriptional cis-elements, and nuclear receptor activation. Taken together, these findings suggest that CA, by regulating targets localized across multiple subcellular compartments, can simultaneously alleviate metabolic oxidative stress, apoptosis imbalance, disrupted gene transcription, and extracellular matrix remodeling in liver tissue, thereby exerting a synergistic, multi-pathway intervention in the complex pathological progression of T2DM-NAFLD comorbidity.

The results of the KEGG enrichment analysis (Figure 6B) show that the targets are significantly enriched in pathways related to glycolipid metabolism, diabetes complications, insulin resistance, and metabolic inflammation. Among these, ‘Metabolic pathways’, ‘Lipid and atherosclerosis’, the ‘AGE-RAGE signaling pathway’, ‘Insulin resistance’, ‘PI3K-Akt’, and the ‘IL-17 signaling pathway’ were identified as core enriched pathways. These pathways serve as key mechanisms through which CA improves glucose and lipid metabolism disorders in T2DM-NAFLD, alleviates insulin resistance, and inhibits AGE glycotoxicity and IL-17-mediated chronic liver inflammation. Additionally, these targets were also enriched in pathways associated with multisystem comorbidities—such as cancer, viral infections, and neurodegenerative diseases—as well as in the hepatic cytochrome P450 drug metabolism pathway. This suggests that, in addition to specifically regulating hepatic glucose and lipid metabolism and ameliorating the core pathological damage of diabetes-associated fatty liver disease, CA also possesses the potential to intervene in multi-organ complications secondary to glucose and lipid dysregulation and to regulate the metabolism of hepatic lipotoxic substances. Together, these findings provide pathway-level evidence for the molecular mechanism underlying CA’s “multifunctional” therapeutic effects on T2DM-NAFLD comorbidity.

### 2.4. Potential Mechanisms of Cinnamaldehyde Intervention in Ochratoxin A-Induced Type 2 Diabetes Mellitus and Non-Alcoholic Fatty Liver Disease Comorbidity

**Figure 7:**
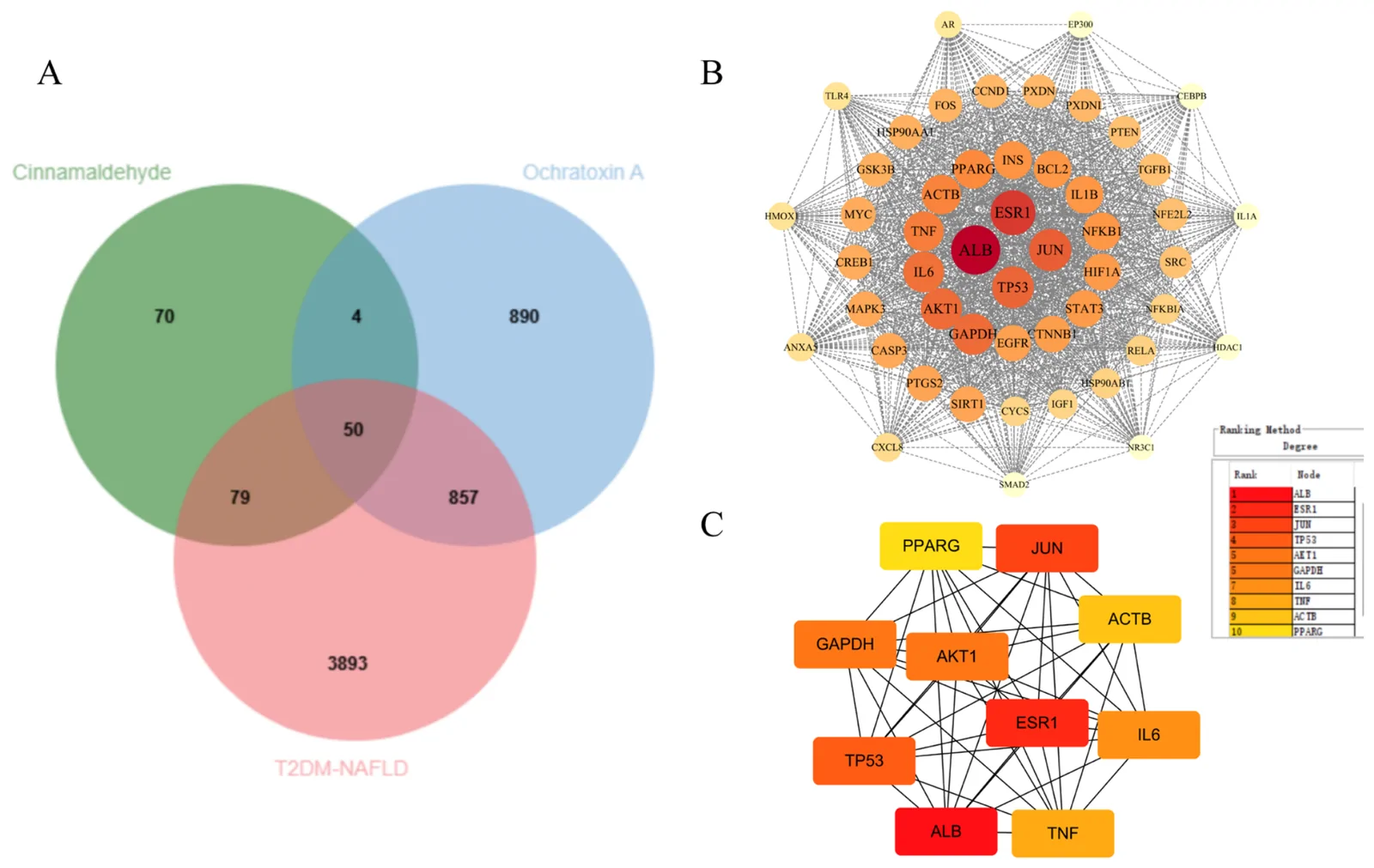
Integrated analysis of network toxicology and network pharmacology. (**A**) A Venn diagram of targets associated with CA, OTA, and T2DM-NAFLD, generated using Venny 2.1. Green, blue, and pink circles represent CA, OTA, and T2DM-NAFLD-associated targets, respectively. (**B**,**C**) A PPI network of the core targets screened using the CytoNCA plugin in Cytoscape 3.10.1, and the top 10 hub genes identified by the Degree algorithm. Nodes represent proteins, while edges represent interactions between proteins. Node color and size are mapped to degree values: a yellow-to-red gradient and increasing node size indicate higher centrality within the network.

**Figure 8:**
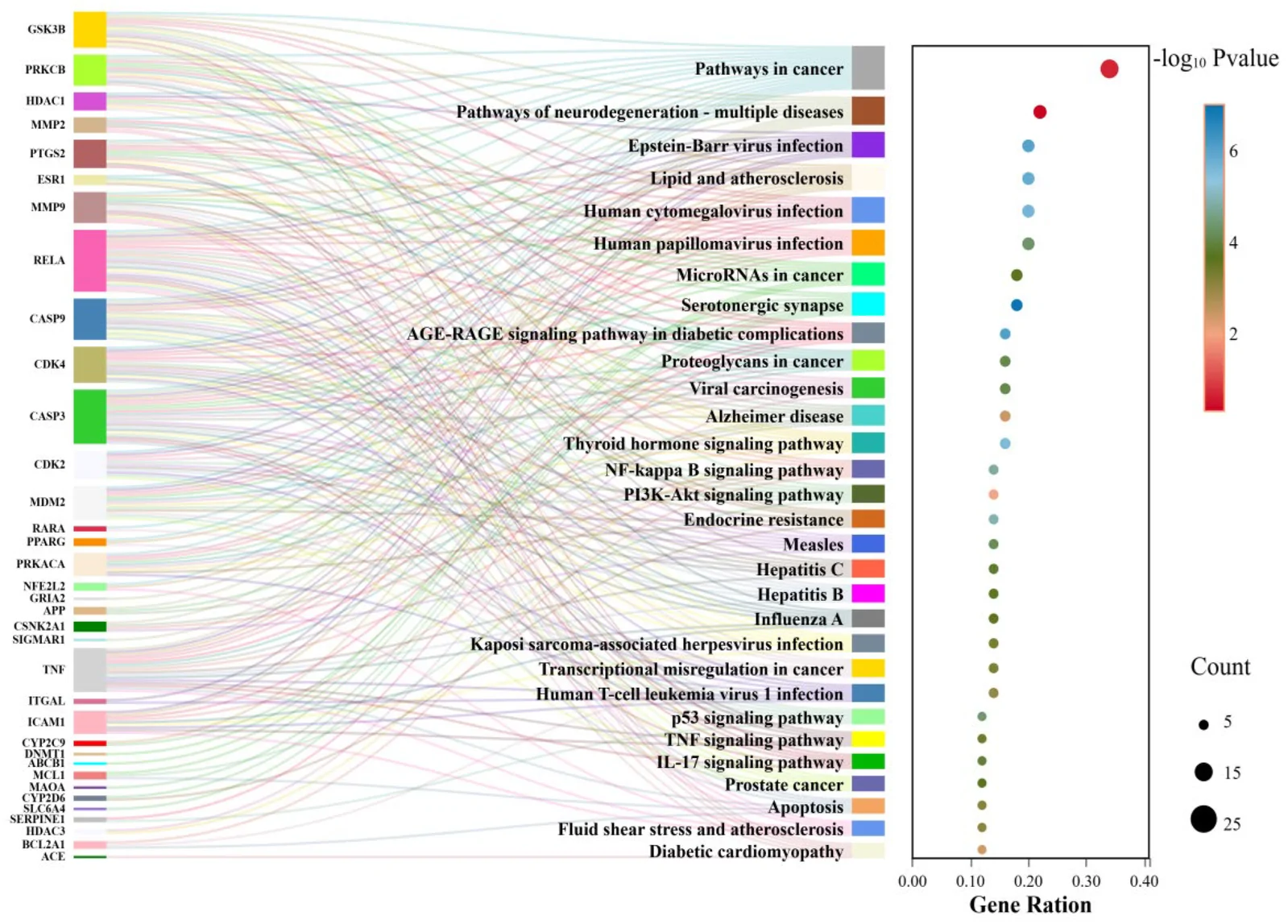
KEGG enrichment analysis from the integrated network toxicology and network pharmacology approach. This figure was generated using the CNSknowall online platform. The color bar on the right represents −log10(*p*-value), with a blue-to-red gradient indicating increasing statistical significance (i.e., decreasing *p*-values). On the left, a Sankey diagram shows core target genes (left nodes) linked to enriched KEGG pathways (right nodes); colored lines represent gene–pathway associations, and line thickness reflects the strength of association or the number of genes involved. The bubble chart on the right displays the gene ratio (the proportion of enriched target genes relative to the total number of input target genes) on the x-axis and KEGG pathway names on the y-axis. Bubble size (Count) indicates the number of enriched genes, and bubble color represents −log10(*p*-value), reflecting enrichment significance.

Based on the results of network toxicology and network pharmacology studies, we found that the mechanism by which CA intervenes in OTA-induced T2DM-NAFLD comorbidity may involve multiple signaling pathways, including the *PI3K-AKT*, *AGE-RAGE* (diabetic complications-associated), *TNF*, and *IL-17* pathways, among others. Further integrative analysis of network toxicology and network pharmacology data identified 50 common target genes (Figure 7A,B), and the top 10 core genes were *ALB*, *TP53*, *ESR1*, *JUN*, *IL6*, *ACTB*, *PPARG*, *GAPDH*, *AKT1*, and *TNF* (Figure 7C). KEGG enrichment analysis (Figure 8) further revealed that these shared genes are primarily enriched in key pathways, including the cancer pathway, the lipid and atherosclerosis pathway, the AGE-RAGE signaling pathway associated with diabetic complications, the PI3K-AKT insulin signaling pathway, the TNF signaling pathway, and the IL-17 signaling pathway.

### 2.5. Analysis of Molecular Docking Results

**Figure 9:**
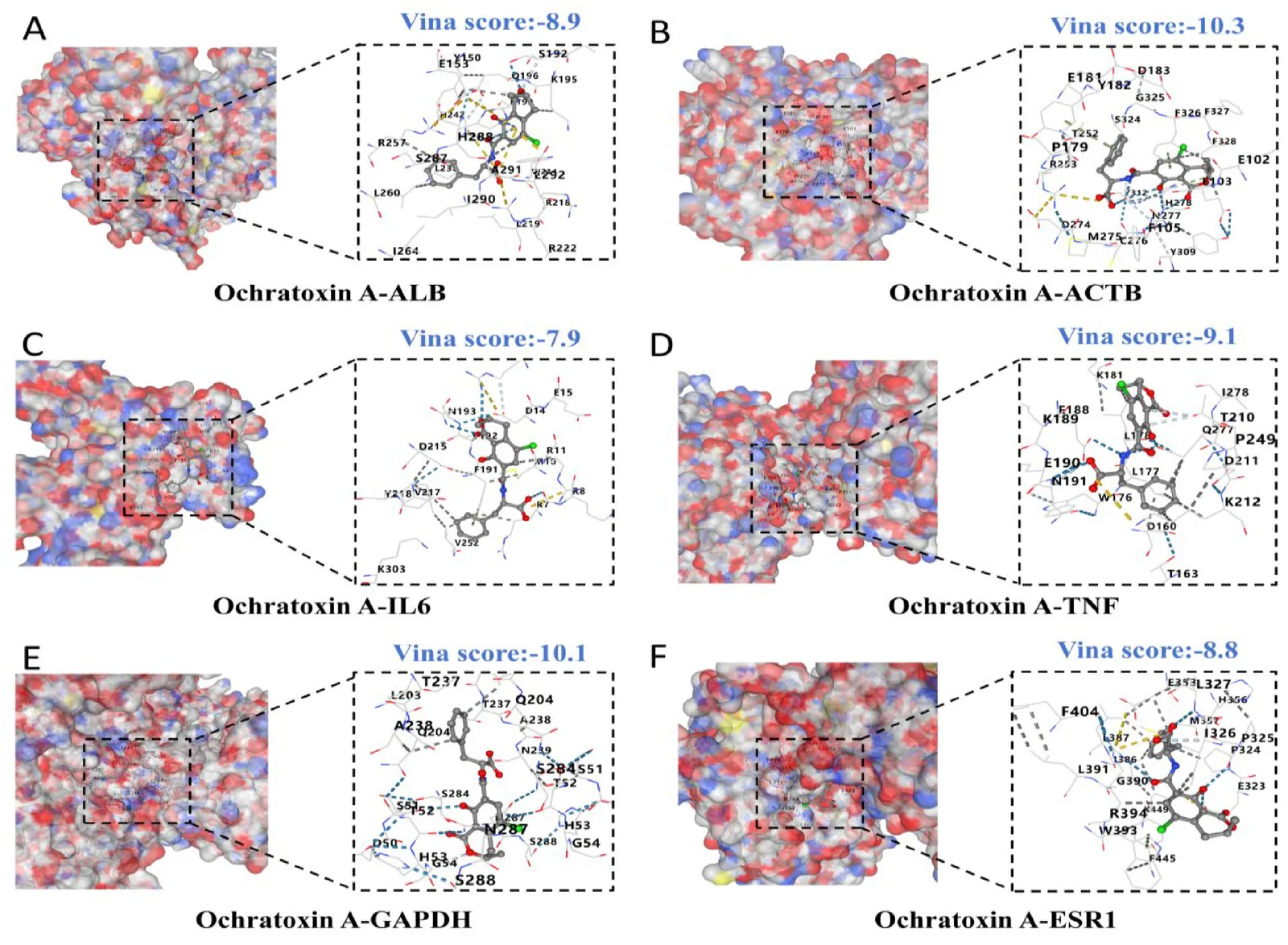
Molecular docking of the toxic compound OTA with its key targets, generated using the CB-DOCK2 online platform.(**A**) OTA-*ALB* (Vina score: −8.9); (**B**) OTA-*ACTB* (Vina score: −10.3); (**C**) OTA-*IL6* (Vina score: −7.9); (**D**) OTA-*TNF* (Vina score: −9.1); (**E**) OTA-*GAPDH* (Vina score: −10.1); (**F**) OTA-*ESR1* (Vina score: −8.8). The zoomed-in views within the dashed boxes display the spatial conformation of the OTA ligand in the binding pockets of the target proteins and the key intermolecular interactions.

**Figure 10:**
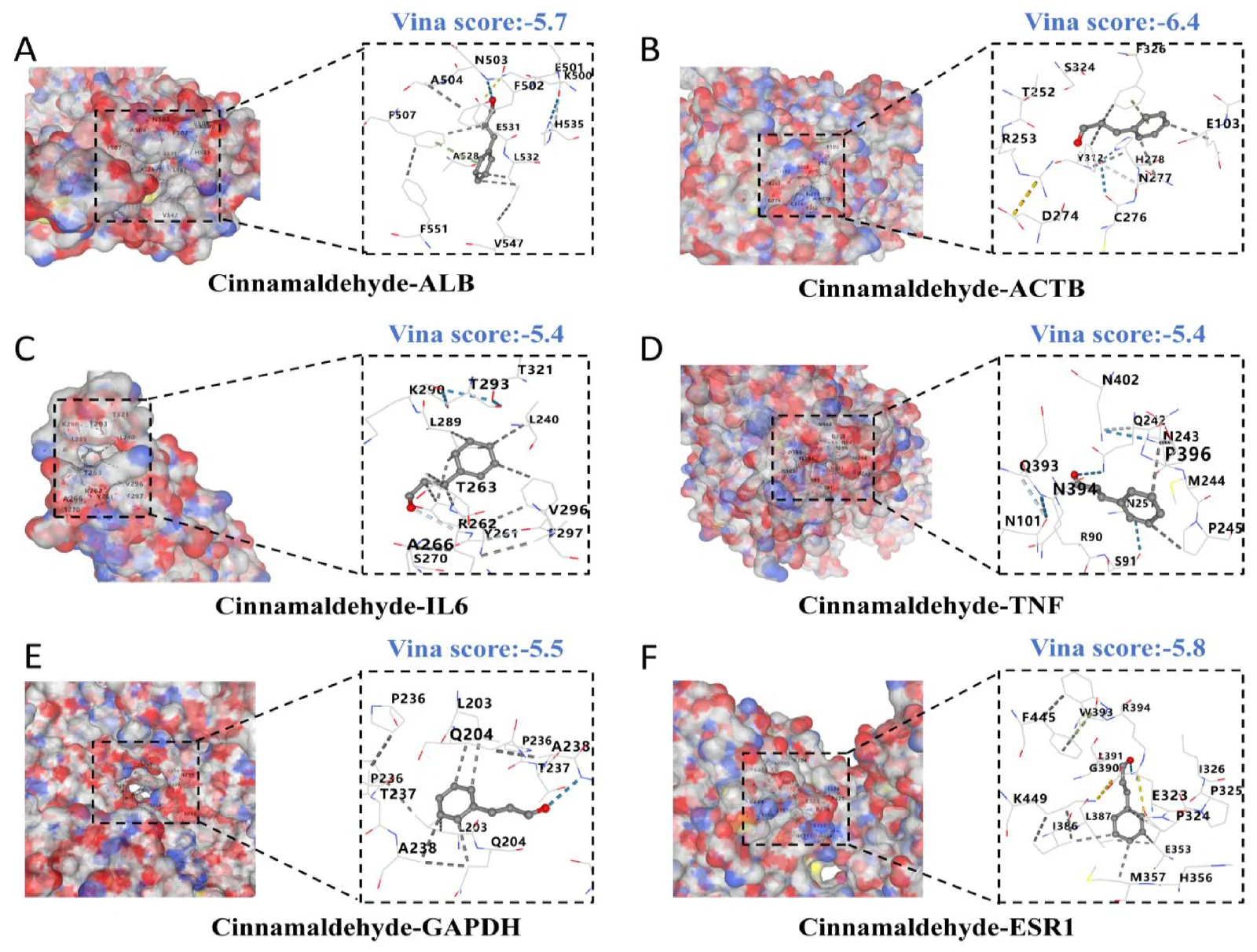
Molecular docking results for CA and the core target, generated using the CB-DOCK2 online platform. (**A**) CA-*ALB* (Vina score: −5.7); (**B**) CA-*ACTB* (Vina score: −6.4); (**C**) CA-*IL6* (Vina score: −5.4); (**D**) CA-*TNF* (Vina score: −5.4); (**E**) CA-*GAPDH* (Vina score: −5.5); (**F**) CA-*ESR1* (Vina score: −5.8). The zoomed-in views within the dashed boxes display the spatial conformation of the CA ligand in the binding pockets of the target proteins and the key intermolecular interactions.

Molecular docking will be performed using the CB-DOCK2 platform [26] on the top 10 core genes identified through comprehensive network toxicology and network pharmacology analyses, and on the core targets of the toxic component OTA and cinnamaldehyde. It is generally accepted that a lower binding energy indicates easier binding of the ligand to the protein and greater complex stability. A binding energy ≤ −5.0 kcal/mol is considered to reflect strong binding activity between the active compound and the target protein. Based on this criterion, six targets with binding energies ≤ −5.0 kcal/mol were selected for further discussion; the remaining four targets, with binding energies > −5.0 kcal/mol, were excluded from this analysis. The molecular docking results (Figure 9 and Figure 10) showed that OTA and cinnamaldehyde both acted on the same set of core targets (*ALB*, *ACTB*, *IL6*, *TNF*, *GAPDH*, and *ESR1*), but with different binding energies. The binding energies of all complexes were below the threshold (≤−5.0 kcal/mol), supporting the reliability of the targets identified by the network analysis. Notably, the binding energies of OTA to the six targets were significantly lower than those of cinnamaldehyde, indicating a higher affinity, which is consistent with the highly toxic nature of OTA.

OTA exhibited markedly strong binding to *ACTB* (−10.3 kcal/mol) and *GAPDH* (−10.1 kcal/mol). *ACTB* and *GAPDH*, encoded by classic housekeeping genes [27], are key regulators of cytoskeletal homeostasis and glycolytic metabolism, respectively [28]. Such exceptionally strong binding suggests that OTA may directly occupy the polymerization interface of *ACTB* or the substrate-binding pocket of *GAPDH*, thereby disrupting basal energy metabolism and cellular structural integrity. This provides a new structural biology perspective on the mechanisms underlying OTA-induced nephrotoxicity and apoptosis. OTA also showed strong binding to *TNF* (−9.1 kcal/mol) and *IL-6* (−7.9 kcal/mol), implying that OTA may directly modulate the secretion of inflammatory cytokines through allosteric mechanisms, independent of upstream signaling cascades. Furthermore, the strong binding of OTA to *ESR1* (−8.8 kcal/mol) provides additional structural evidence supporting its potential role as an endocrine disruptor. In contrast, CA showed binding energies close to the −5.0 kcal/mol threshold for all targets. This relatively weak affinity does not imply a lack of efficacy; rather, it suggests that CA does not act as a competitive antagonist by occupying active sites with high affinity. Instead, it may modulate protein function through weak multi-target synergistic effects or by binding to allosteric sites. This indicates that, as a mild antioxidant and anti-inflammatory agent, cinnamaldehyde may exert protective effects without causing severe cytotoxicity.

In summary, the molecular docking results indicate a marked difference in binding energy between OTA and CA across the core targets. The toxic effects of OTA may involve a “strong binding–direct interference” mechanism, while the protective effects of CA may involve a “weak binding–indirect regulation” mechanism. Consequently, CA may be unable to counteract OTA toxicity through simple competitive displacement; instead, its detoxification mechanism may rely on the upregulation of downstream metabolic enzymes or the induction of target protein degradation.

## 3. Discussion and Conclusions

Type 2 diabetes and non-alcoholic fatty liver disease are two highly prevalent metabolic disorders that often coexist. Their comorbidity involves extremely complex mechanisms and presents considerable challenges for clinical intervention [29], as multiple intertwined pathophysiological pathways—including insulin resistance, lipid metabolism abnormalities, chronic low-grade inflammation, and oxidative stress—are involved [30]. Insulin resistance serves as a common pathophysiological link between these two diseases: it not only drives de novo lipid synthesis and accumulation in the liver, but also impairs glucose uptake and utilization in peripheral tissues, thereby establishing a vicious cycle [19]. Disturbances in hepatic glucose and lipid metabolism constitute the core pathophysiological basis of this comorbidity. Exposure to environmental toxins is also considered a major contributing factor, with particular emphasis on the widely prevalent mycotoxin OTA, which has been shown to significantly accelerate the onset and progression of T2DM-NAFLD comorbidity by inducing hepatic insulin resistance, disrupting glucose and lipid metabolism homeostasis, and triggering chronic low-grade inflammation [31]. CA, a natural bioactive compound, exhibits multiple pharmacological activities, including antioxidant and anti-inflammatory effects, the regulation of glucose and lipid metabolism, and improvement in insulin resistance [32], demonstrating great potential for multi-target modulation of metabolic disorders. However, the potential molecular mechanisms underlying CA intervention in OTA-induced T2DM-NAFLD comorbidity remain to be systematically elucidated.

Existing network pharmacology studies have largely focused on the direct effects of a single disease or drug, while the integration of toxicological mechanisms of environmental toxins with the therapeutic mechanisms of natural components in traditional Chinese medicine remains relatively unexplored. To elucidate the mechanism by which CA exerts a protective effect against OTA-induced T2DM-NAFLD comorbidity, this study first performed separate network toxicology and network pharmacology analyses. The former investigated the potential mechanisms underlying OTA-induced T2DM-NAFLD comorbidity, while the latter examined how CA exerts its protective effect. We then conducted a comprehensive integrative analysis to clarify the mechanism by which CA intervenes in this comorbidity. Multiple databases—including PubChem, CTD, TargetNet, SEA, GeneCards, and STRING—were integrated to identify and analyze relevant targets. Systematic network analysis of these targets was performed using Cytoscape 3.10.1 software, followed by GO functional annotation and KEGG pathway enrichment analysis of the core targets via the CNSknowall platform. Finally, molecular docking studies were carried out using the CB-DOCK2 platform to systematically investigate the potential molecular mechanisms underlying CA intervention in OTA-induced T2DM-NAFLD comorbidity.

Through comprehensive analysis of network toxicology and network pharmacology findings, the core genes involved in CA intervention in OTA-induced T2DM-NAFLD comorbidity were identified as follows: *ALB*, *TP53*, *ESR1*, *JUN*, *IL6*, *ACTB*, *PPARG*, *GAPDH*, *AKT1*, and *TNF*. The following discussion will focus on these core targets, along with genes exhibiting high docking binding energies with CA and OTA, and the core enriched pathways.

*ALB* is the most abundant transport protein in serum and plays a key role in maintaining plasma colloid osmotic pressure and transporting various endogenous and exogenous substances, such as fatty acids, hormones, and drugs [33]. In the context of T2DM-NAFLD comorbidity, *ALB* exhibits a complex dual role. On the one hand, hypoalbuminemia is an independent risk factor for impaired liver function and disease progression [34]. Clinical studies have confirmed that reduced serum albumin levels in patients with NAFLD are significantly associated with the severity of liver fibrosis and serve as independent predictors of non-alcoholic steatohepatitis and advanced fibrosis [35,36]. Furthermore, reduced albumin levels are also associated with an increased risk of developing T2DM. One study found that baseline serum albumin levels were negatively correlated with the risk of T2DM, and that a dynamic decline in albumin levels significantly increased the risk of T2DM onset [37]. In patients with T2DM and NAFLD, low albumin levels are closely associated with more severe hepatic steatosis, inflammation, and fibrosis progression, and can serve as an important biomarker for assessing disease severity and prognosis [38,39]. On the other hand, the function of *ALB* is not limited to its concentration; its binding capacity has emerged as a valuable indicator in disease diagnosis [40]. Studies have shown that in patients with NAFLD, albumin binding capacity is significantly reduced and positively correlated with liver fibrosis indices, suggesting that impaired albumin binding function may serve as a novel biomarker for early liver damage and disease progression [41]. Molecular docking results show that the binding energy between OTA and *ALB* is −8.9 kcal/mol, indicating strong binding affinity. This may interfere with albumin’s normal transport of fatty acids, bilirubin, drugs, and various metabolites, thereby exacerbating the metabolic burden and toxic damage to the liver. The binding energy between CA and *ALB* is −5.7 kcal/mol. CA may partially reverse the OTA-induced disruption of *ALB* function through competitive binding or allosteric regulation, thereby restoring some degree of normal transport function and alleviating metabolic disorders.

As a core component of the cytoskeleton, *ACTB* not only maintains cell morphology but also participates in key biological processes such as cell migration, signal transduction, and metabolic regulation [42]. In the pathological progression of T2DM-NAFLD comorbidity, abnormal *ACTB* expression in hepatocytes is closely associated with steatosis and impaired insulin signaling. Molecular docking results show that OTA binds to *ACTB* with a binding energy of −10.3 kcal/mol, indicating strong binding affinity. This strong interaction may directly compromise the cytoskeletal integrity of hepatocytes and pancreatic β-cells, leading to abnormal cell morphology, functional impairment, and even apoptosis [43]. Disruption of the hepatocyte cytoskeleton further impairs lipid metabolism and detoxification, while damage to the pancreatic β-cell cytoskeleton directly affects insulin synthesis and secretion [44], thus playing a bridging role in the pathogenesis of T2DM-NAFLD comorbidity. The binding energy of CA to *ACTB* is −6.4 kcal/mol, suggesting a relatively mild interaction that may help preserve cellular structure. As a natural bioactive compound, CA exhibits antioxidant, anti-inflammatory, and metabolic regulatory effects. In hepatocytes, CA may alleviate OTA-induced cytoskeletal damage by partially competing with OTA for *ACTB* binding or by indirectly stabilizing the cytoskeletal network through the activation of antioxidant pathways such as Nrf2 [45]. Additionally, CA may protect hepatocytes from metabolic stress by regulating autophagy to promote the clearance of damaged cytoskeletal components.

*IL6* and *TNF* are the most critical pro-inflammatory factors in T2DM-NAFLD comorbidity, serving as the central hub linking metabolic disorders and inflammatory responses [46]. In this comorbidity, hepatic steatosis and insulin resistance activate Kupffer cells and infiltrating macrophages in the liver, leading to the secretion of large amounts of *IL6* and *TNF* [47]. These cytokines not only directly damage pancreatic β-cells, resulting in insufficient insulin secretion, but also amplify inflammatory cascades in the liver and adipose tissue by activating downstream signaling pathways such as JAK/STAT and NF-κB, thereby exacerbating insulin resistance and hepatocyte damage [48]. Studies have shown that in patients with T2DM complicated by NAFLD, circulating levels of *IL6* and *TNF* are significantly elevated and positively correlated with disease severity [49]. The binding energies of OTA to *IL6* and *TNF* are −9.1 and −7.9 kcal/mol, respectively, suggesting that OTA may exacerbate the inflammatory response by stabilizing the active conformations of these pro-inflammatory factors or enhancing their receptor binding. CA exhibits binding energies of −5.4 and −5.5 kcal/mol for *IL6* and *TNF*, respectively. Its relatively weak binding affinity for *IL6* may allow for a “partial antagonistic” effect in vivo: it does not completely abolish the physiological functions of *IL6* but effectively reduces the pathological effects resulting from its excessive activation. This regulatory mechanism is consistent with the traditional pharmacological properties of CA—namely, “warming the middle burner, dispelling cold, and harmonizing qi and blood”—suggesting that it may alleviate inflammatory damage while maintaining immune homeostasis through a multi-target, weak interaction network. Therefore, *IL6* and *TNF* serve as key nodes in the anti-inflammatory interplay between CA and OTA, and represent core targets for CA intervention in T2DM-NAFLD comorbidity.

*GAPDH* is a key rate-limiting enzyme in the glycolytic pathway, responsible for converting D-glyceraldehyde-3-phosphate to 1,3-diphosphoglycerate while reducing NAD^+^ to NADH [50]. It is a central component of cellular energy metabolism. In patients with T2DM-NAFLD, energy metabolism in the liver and pancreatic β-cells is already disrupted. With a binding energy of −10.1 kcal/mol, OTA may directly inhibit *GAPDH* activity, leading to impaired glycolysis and reduced ATP production, thereby contributing to T2DM-related metabolic disorders [51]. Inhibition of *GAPDH* activity also affects other NADH-dependent metabolic pathways, such as fatty acid synthesis and oxidation, further disrupting hepatic lipid metabolism homeostasis [52]. CA exhibits weaker binding affinity for *GAPDH*, with a binding energy of −5.4 kcal/mol; it may mitigate oxidative stress-induced liver damage by partially modulating *GAPDH* activity, while preserving basal glucose metabolism.

*ESR1*, a key member of the nuclear receptor superfamily [53], exhibits significant gender-specific roles in metabolic regulation and mediates various physiological functions of estrogen, including regulation of lipid metabolism, anti-inflammatory effects, and protection against oxidative stress [54]. In the comorbidity of T2DM and NAFLD, estrogen is generally considered protective, alleviating hepatic steatosis and inflammation [55]. In the liver, activation of *ESR1* improves the pathological progression of non-alcoholic fatty liver disease through multiple mechanisms. Molecular docking results show that OTA binds to *ESR1* with a binding energy of −8.8 kcal/mol, indicating high affinity. As a mycotoxin, OTA has a structure similar to that of estrogen and can competitively bind to the ligand-binding domain of *ESR1*, leading to receptor conformational changes and thereby inhibiting the transcriptional activity of downstream target genes [56]. This interference may attenuate the protective effects of estrogen and aggravate disease progression. Notably, OTA’s interference with *ESR1* is gender-dependent and may be more pronounced in women, as *ESR1* expression levels are higher in female livers, and the estrogen signaling pathway plays a more dominant role in metabolic homeostasis [57]. Therefore, OTA exposure may induce more severe metabolic disorders in women—including hepatic steatosis, insulin resistance, and chronic inflammation—by disrupting *ESR1* function. The binding energy of CA to *ESR1* is −5.8 kcal/mol, suggesting that CA may regulate downstream *ESR1* signaling through indirect pathways, which may promote its expression. This could, to some extent, improve lipid metabolism, insulin sensitivity, and inflammatory status, thereby exerting a protective effect.

KEGG enrichment analysis integrating network pharmacology, network toxicology, and their combined data revealed that the identified targets were primarily enriched in core signaling pathways, including cancer, lipid and atherosclerosis, *AGE-RAGE*, *PI3K-Akt* insulin signaling, *TNF signaling*, and *IL-17 signaling*.

In the comorbidity of T2DM and NAFLD, abnormal activation of multiple key signaling pathways constitutes the core molecular basis driving disease progression. OTA, a common pollutant, can exacerbate the condition by disrupting these pathways through multi-target mechanisms, whereas CA can specifically reverse these pathway abnormalities and exert a protective effect. Cancer-related signaling pathways and the lipid/atherosclerosis pathway are key systems regulating metabolic disorders and organ damage in this disease. Among these, the *PI3K/Akt/mTOR* and *Ras/Raf/MEK/ERK* signaling cascades are abnormally activated in T2DM-NAFLD and play roles in regulating cell proliferation, apoptosis, and metabolism. Hepatic insulin resistance and lipid accumulation can sustainably activate these oncogenic signaling pathways, driving abnormal hepatocyte proliferation and liver fibrosis [58]. OTA can promote hepatic inflammation and cell proliferation by activating core transcription factors such as NF-κB and AP-1, thereby accelerating the progression from hepatic steatosis to non-alcoholic steatohepatitis [59]. Additionally, by inducing oxidative stress, OTA continuously activates the *PI3K/Akt* pathway, creating a vicious cycle of sustained pathway activation. In the lipid and atherosclerosis pathway, the lipid metabolism disorders triggered by T2DM-NAFLD serve as precursors to cardiovascular disease [60]; hepatic lipid accumulation and insulin resistance disrupt lipoprotein secretion homeostasis, thereby increasing cardiovascular risk. OTA can upregulate sterol regulatory element-binding protein-2 and low-density lipoprotein receptors, promoting hepatic cholesterol synthesis and peripheral lipid deposition, and activating vascular inflammatory responses [24], thus further exacerbating cardiovascular damage in patients with T2DM-NAFLD comorbidity.

CA can bidirectionally regulate the aforementioned pathways: it blocks oncogenic signaling by inhibiting the abnormal activation of *PI3K/Akt* and *NF-κB* [61], thereby alleviating liver fibrosis and protecting pancreatic β-cells. Meanwhile, by activating the liver X receptor and peroxisome proliferator-activated receptor α, it promotes cholesterol reverse transport and fatty acid oxidation, thus improving the plasma lipid profile, inhibiting lipid deposition and atherosclerotic lesions, and ultimately delaying the progression of T2DM-NAFLD comorbidity.

Disruptions in the *AGE-RAGE*, *PI3K-Akt*, insulin, and inflammation-related signaling pathways constitute key mechanisms by which OTA mediates metabolic imbalances and inflammatory damage in T2DM-NAFLD. These pathways are interconnected, forming a complex pathogenic network. Hyperglycemia and oxidative stress continuously activate the *AGE-RAGE* pathway; upon binding to their receptors, AGEs trigger downstream *NF-κB* and *MAPK* signaling, mediating inflammatory and fibrotic responses [62]. OTA can exacerbate oxidative stress, promote AGE production, and upregulate RAGE expression, thereby enhancing pathway activation and inducing liver and kidney dysfunction, thus worsening T2DM-NAFLD comorbidity. The *PI3K-Akt* insulin signaling pathway is central to insulin action; impaired insulin signaling leads to reduced glucose uptake, increased hepatic glucose output, and hepatic lipid accumulation [63,64]. OTA can inhibit phosphorylation of IRS-1, PI3K, and Akt, thereby blocking insulin signaling and aggravating glucose–lipid metabolic disorders and hepatic insulin resistance. The *TNF* and *IL-17* pathways are central to mediating chronic inflammation in T2DM-NAFLD. The *TNF* pathway induces the release of large amounts of inflammatory cytokines by activating NF-κB and MAPK, promoting insulin resistance and hepatocyte apoptosis [65]. The *IL-17* pathway mediates neutrophil infiltration and liver fibrosis [66], and the two pathways act synergistically to exacerbate liver damage. OTA can simultaneously activate both inflammatory pathways, creating an amplified inflammatory cascade that promotes the progression of hepatic steatosis to NASH and fibrosis [67].

CA can intervene in the aforementioned pathogenic pathways through multiple targets. It alleviates liver and kidney damage by scavenging reactive oxygen species, inhibiting AGE formation, and suppressing RAGE signaling [68]; restores the insulin signaling pathway by increasing Akt phosphorylation levels, thereby improving glucose and lipid metabolism abnormalities; and simultaneously reduces the expression of inflammatory factors such as TNF-α and IL-17, breaking the vicious cycle of inflammation. Through this multi-pathway interactive network, CA comprehensively improves the pathological damage associated with OTA-induced T2DM-NAFLD comorbidity.

CA is a lipophilic, small-molecule aldehyde with a molecular weight of approximately 132.16 g/mol and an octanol–water partition coefficient (logP) of approximately 1.9. Owing to its balanced lipophilic and hydrophilic properties, it readily crosses the gastrointestinal barrier, penetrates the cell membranes of hepatocytes and pancreatic β-cells [69], and distributes to target organs of OTA, such as the liver and kidneys. OTA is a weakly acidic mycotoxin with a molecular weight of approximately 403.81 g/mol and a logP of 4.74. It is more lipophilic than CA, tends to accumulate in adipose tissue, and exhibits persistent and cumulative toxicity. In contrast, CA has a half-life of only approximately one hour [70] and is rapidly cleared; while this rapid elimination helps prevent accumulation, it also limits its long-term protective effect, a limitation that must be addressed through optimization of the dosing regimen. Regarding protective mechanisms, the α,β-unsaturated aldehyde group of CA confers electrophilicity [71], enabling it to form covalent bonds with thiol or amino groups on proteins or DNA to modulate signaling pathways [72]. For instance, it may block the *NF-κB* pathway to inhibit OTA-induced inflammation or activate the *PI3K/AKT* pathway to counteract oxidative stress and apoptosis, thereby attenuating the toxic cascade triggered by OTA at multiple stages. However, this same electrophilicity also poses a dose-dependent risk: inappropriate dosing may cause non-specific reactions or even additive toxicity with OTA. Therefore, although the high bioavailability and ability of CA to cross biological barriers suggest its potential to protect target organs, the dose must be precisely controlled to balance protective effects against potential adverse reactions.

As a dietary supplement [73], the safety of CA is of paramount importance. CA has been designated as Generally Recognized as Safe (GRAS) by the U.S. Food and Drug Administration (FDA); it occurs naturally in a variety of foods, and its Acceptable Daily Intake (ADI) is approximately 0.5–1.0 mg/kg body weight [74]. Studies have shown that CA exhibits significant chemopreventive potential at low doses [75]; for example, it protects against myocardial ischemia/hypoxia-induced injury by activating the *PI3K/AKT* signaling pathway, thereby inhibiting oxidative stress and apoptosis [61]. The anti-inflammatory activity of CA has also been confirmed; it can suppress lipopolysaccharide (LPS)-induced inflammation in osteoarthritis-associated synovial fibroblasts by blocking the *TLR4/MyD88* signaling pathway and alleviate chondrocyte inflammation and cartilage degeneration by inhibiting *NF-κB* pathway activation [76]. To improve the stability and bioavailability of CA, various delivery systems have been developed, including microemulsions, poly(lactic-co-glycolic acid) (PLGA) nanoparticles, and chitosan–alginate bilayer microcapsules [77]. Microemulsions can increase its oral bioavailability approximately 2.5-fold [78]; PLGA nanoparticles can enhance its antifungal and anti-biofilm efficacy, producing potent effects even at lower concentrations of the active compound [79]. However, the safety of CA is dose-dependent; high doses may cause hepatotoxicity or gastrointestinal irritation [80]. Its α,β-unsaturated aldehyde group is the key structural feature responsible for its antioxidant and anti-inflammatory activities, but it is also the source of its potential toxicity. Therefore, when CA is used as a dietary supplement, the dosage must be strictly controlled to ensure that its beneficial biological effects are realized while avoiding potential adverse reactions.

The results of this study indicate that the integration of network toxicology and network pharmacology not only establishes a preliminary theoretical framework for elucidating the toxicity mechanisms of common food and pharmaceutical contaminants, but also, to a certain extent, clarifies the potential mechanism by which cinnamaldehyde—a natural bioactive component of traditional Chinese medicine—intervenes in OTA-induced T2DM-NAFLD comorbidity. The OTA toxicity targets and CA intervention targets identified in this study may provide new avenues for preventing OTA-induced metabolic toxicity, while also offering novel insights into the prevention of T2DM-NAFLD comorbidity.

This study has several limitations. First, both the network toxicology and network pharmacology results rely on predictive data from public databases; the target sites of CA and the toxicity targets of OTA may include false positives, multifunctional genes, or overly broad coverage. Although this bias can be partially mitigated through cross-analysis of multiple databases and intersection screening of targets, it cannot be completely eliminated. Second, molecular docking simulations merely reflect the spatial binding affinity between ligands and receptors; they cannot directly demonstrate the activation, inhibition, or functional regulation of these targets by CA in cells or living organisms. Third, this study has not yet been validated experimentally using cell cultures, animal models, or clinical samples. Therefore, whether CA can actually intervene in OTA-induced insulin resistance, hepatic steatosis, and metabolic disorders via the predicted signaling pathways—such as *PI3K-Akt*, *AGE-RAGE*, and *TNF* signaling—remains to be verified by subsequent experiments. Future research should prioritize the use of cellular and animal models of type 2 diabetes combined with non-alcoholic fatty liver disease, employing molecular biology techniques to verify changes in the expression of key targets and the modulatory effects of CA, to further validate the computational predictions of this study.

## 4. Materials and Methods

### 4.1. Identification of Disease Targets for Type 2 Diabetes Mellitus and Non-Alcoholic Fatty Liver Disease

We searched the CTD (https://ctdbase.org/, accessed on 6 June 2026) and the GeneCards database (https://www.genecards.org/, accessed on 6 June 2026) using the keywords ‘Type 2 Diabetes Mellitus’ and ‘Non-alcoholic Fatty Liver Disease’. To ensure that the identified genes are highly associated with the pathophysiological effects of T2DM and NAFLD, the relevance score threshold in GeneCards was set to >0. After deduplication and organization, the two target libraries—comprising the disease-associated targets and the OTA gene targets—were merged and deduplicated to construct an intersection target library. The Venny 2.1 website (https://bioinfogp.cnb.csic.es/tools/venny/, accessed on 7 June 2026) was used to generate a Venn diagram of the intersection targets. The resulting set of target genes was then imported into the STRING database (v12.0, https://string-db.org/, accessed on 7 June 2026), with an interaction confidence threshold set to ≥0.4 and isolated nodes hidden, to construct a PPI network. The PPI network data were imported into Cytoscape 3.10.1 software (Cytoscape Consortium, San Diego, CA, USA; https://cytoscape.org/index.html, accessed on 10 October 2025), and the CytoHubba plugin was utilized to perform multidimensional network topological analysis in order to systematically identify key hub genes located at the control core of the network. The topological parameters analyzed and their biological implications are as follows: Betweenness Centrality: Quantified as the frequency with which all shortest paths in the network pass through that node. A higher value indicates that the node plays a more significant role as a ‘bridge’ and a bottleneck for information transmission between different functional modules. Closeness Centrality: Measured as the reciprocal of the average shortest distance from the node to all other nodes in the network. A higher value indicates that the node is topologically closer to the global center, allowing information to propagate rapidly via shorter paths. Degree Centrality: The number of neighbors directly connected to a node, which intuitively reflects its local connectivity scale. Eigenvector Centrality: Builds upon degree centrality by incorporating weightings based on the importance of neighboring nodes; that is, being connected to high-scoring nodes results in a higher score for the node itself. LAC Score: Quantifies the connectivity density of the local subnet formed by a node’s neighbors, indicating whether the node is embedded within a tightly knit functional complex. Network Centrality: The average of the degree values of all a node’s neighbors; this is used to assess whether the local network microenvironment in which the node resides is enriched with other highly connected nodes. By selecting a combination of the above multidimensional parameters, we comprehensively measure a node’s topological importance from multiple complementary perspectives—including local versus global, direct versus indirect, and independent importance versus neighborhood dependence—thereby avoiding the information gaps and biases that may arise from relying on a single metric. Threshold determination: To ensure an objective and unbiased selection, we calculated the values of the six parameters mentioned above within the PPI network and uniformly adopted the median of each parameter as the cut-off threshold: any node that does not fall below the corresponding median value across all six dimensions is considered to be topologically more important than more than half of the nodes in the network and is defined as a core target. All screening of potential targets in this paper utilizes the median of the aforementioned multidimensional parameters as the cut-off threshold.

### 4.2. Collection of Toxicological Data on Ochratoxin A-Induced Type 2 Diabetes Mellitus and Non-Alcoholic Fatty Liver Disease Complications

#### 4.2.1. Physical and Chemical Properties of Ochratoxin A and Collection of Toxicity Parameters

We retrieved the SMILES sequence for ochratoxin A from the PubChem database (https://pubchem.ncbi.nlm.nih.gov/, accessed on 5 June 2026), and then input it into the ADMETlab 3.0 (https://admetlab3.scbdd.com/, accessed on 5 June 2026) and ProTox 3.0 (https://tox.charite.de/protox3/, accessed on 5 June 2026) databases to obtain the prediction results.

#### 4.2.2. Ochratoxin A Target Prediction

Using “Ochratoxin A” as the keyword, we retrieved the chemical structure and SMILES representation of this compound from the PubChem database (https://pubchem.ncbi.nlm.nih.gov/, accessed on 5 June 2026). Data on potential OTA targets were obtained from the CTD (https://ctdbase.org/, accessed on 6 June 2026) and GeneCards (https://www.genecards.org/, accessed on 6 June 2026). Following integration of the search results and the removal of duplicates, a dataset of potential OTA targets was established; all targets were standardized using UniProt IDs.

#### 4.2.3. Construction of Ochratoxin A-Disease Intersection Targets and Protein–Protein Interaction Networks

The two target libraries—disease-related targets and OTA-related gene targets—were merged and deduplicated to construct an intersection target library. A Venn diagram of the intersection targets was generated using the Venny 2.1 website (https://bioinfogp.cnb.csic.es/tools/venny/, accessed on 7 June 2026). The intersection targets were then imported into the STRING database (v12.0, https://string-db.org/, accessed on 8 June 2026) to build a PPI network, with the species set to homo sapiens and the interaction score threshold set at ≥0.4. The STRING results were subsequently imported into Cytoscape software for visualization and topological analysis, including calculation of network parameters and visualization of the PPI network. The screening criteria for core targets of OTA-induced T2DM-NAFLD comorbidity included node degree, closeness centrality, betweenness centrality, eigenvector centrality, network structure, and LAC score. Finally, the cytohubba plugin in Cytoscape was used to predict the top 10 key genes based on the Degree algorithm.

#### 4.2.4. Gene Ontology Analysis and Kyoto Encyclopedia of Genes and Genomes Pathway Analysis of Ochratoxin A and Disease Targets

The DAVID database (https://davidbioinformatics.nih.gov/, accessed on 11 June 2026) was used to perform GO functional and KEGG pathway enrichment analyses on the intersection of target genes, identifying the top 10 GO terms and the top 20 KEGG pathways. Finally, the results were visualized on the CNSknowall platform (https://cnsknowall.com/, accessed on 11 June 2026).

### 4.3. Collection of Pharmacological Data on Cinnamaldehyde for Treatment of Type 2 Diabetes Mellitus and Non-Alcoholic Fatty Liver Disease Complications

#### 4.3.1. Target Prediction for Cinnamaldehyde

Using “Cinnamaldehyde” as the keyword, we retrieved the chemical structure and SMILES representation of this compound from the PubChem database. Data on potential targets for CA were obtained from the TargetNet database (http://targetnet.scbdd.com/, accessed on 6 June 2026) and the SEA database (https://sea.bkslab.org/, accessed on 6 June 2026). After integrating the search results from these databases and removing duplicates, a dataset of potential CA targets was established. All targets were subsequently standardized using UniProt IDs.

#### 4.3.2. Construction of Cinnamaldehyde–Disease Overlap Targets and Protein–Protein Interaction Networks

The two target libraries—disease-related gene targets and CA gene targets—were merged and deduplicated to construct an intersection target library. Subsequently, the Venny 2.1 website (https://bioinfogp.cnb.csic.es/tools/venny/, accessed on 7 June 2026) was used to generate a Venn diagram of the intersection between CA targets and disease-related targets. The intersection targets were then imported into the STRING database to construct a PPI network, with the species set to homo sapiens and an interaction score threshold of ≥0.4 used to generate the PPI network diagram. The STRING database (https://string-db.org/, accessed on 8 June 2026) results were imported into Cytoscape software for visualization and topological analysis, enabling the calculation of network properties and the generation of a PPI network diagram. The screening criteria for core targets of CA intervention in T2DM-NAFLD comorbidity were node degree, closeness centrality, betweenness centrality, eigenvector centrality, network structure, and LAC score. Finally, the cytohubba plugin in Cytoscape was used to predict the top 10 key genes based on the Degree algorithm.

#### 4.3.3. Gene Ontology Analysis and Kyoto Encyclopedia of Genes and Genomes Pathway Analysis

GO functional and KEGG pathway enrichment analyses were performed on the intersection of target genes using the DAVID database (https://davidbioinformatics.nih.gov/, accessed on 11 June 2026), identifying the top 10 GO terms and the top 20 KEGG pathways for each category. Finally, the results were visualized on the CNSknowall (https://cnsknowall.com/, accessed on 11 June 2026) platform.

### 4.4. Integrated Analysis of Network Toxicology and Network Pharmacology

The potential mechanisms by which CA intervenes in OTA-induced T2DM-NAFLD comorbidity were initially elucidated using network toxicology and network pharmacology approaches. To achieve a more precise understanding, we integrated these two approaches for a comprehensive analysis. Venn diagrams were used to identify overlapping targets among OTA, CA, and T2DM-NAFLD comorbidity. Subsequently, PPI network analysis, top 10 core gene analysis, and KEGG pathway analysis were conducted to explore the underlying mechanisms of action.

### 4.5. Molecular Docking

We downloaded the SDF-format files for ochratoxin A and CA from the PubChem database (https://pubchem.ncbi.nlm.nih.gov/, accessed on 5 June 2026), and downloaded the PDB-format files for the core genes ALB, GAPDH, IL6, TNF, ACTB, and ESR1 from the RCSB PDB database (https://www.rcsb.org/, accessed on 18 June 2026). Then, we used the CB-DOCK2 platform (http://183.56.231.194:8001/cb-dock2/index.php, accessed on 18 June 2026) to perform molecular docking calculations between the core genes identified through a comprehensive analysis of network toxicology and network pharmacology and the top 10 core genes associated with OTA and CA in order to determine binding energies.

### 4.6. Statistical Analysis

Data processing, network topology analysis, and statistical tests for this study were all performed on the respective platforms. To calculate the topological properties of the PPI network, the CytoHubba plugin in Cytoscape 3.10.1 was used to extract six topological parameters for each node: betweenness centrality, closeness centrality, degree centrality, eigenvector centrality, LAC score, and network. To ensure objectivity in core target selection, the median value of each of these six parameters was adopted as a uniform cut-off threshold; nodes with values equal to or greater than the corresponding medians across all six parameters were defined as potential targets.

The initial data for both GO and KEGG pathway enrichment analyses were processed using the DAVID database. DAVID uses the EASE Score—a modified version of the exact hypergeometric test—as its core significance algorithm and applies the Benjamini–Hochberg (BH) method for multiple testing correction. The results directly output by the DAVID platform include *p*-values, as well as Bonferroni-, Benjamini-, and FDR-corrected values. In this study, a Benjamini-corrected *p*-value (i.e., FDR) < 0.05 was strictly set as the threshold for significant enrichment, and the top 10 most significant GO terms and the top 20 most significant KEGG pathways were selected accordingly. For visualization, the raw *p*-values of the significant entries were transformed into −log_10_(*p*-value) to better illustrate the hierarchy of significance. Bubble charts and bar charts were generated using the CNSknowall platform. Specific access dates and version information for all databases and software are provided in Section 4 above.

## Data Availability

All data generated or analyzed during this study are available within the article.

## Abbreviations

Cinnamaldehyde: CA; type 2 diabetes mellitus: T2DM; non-alcoholic fatty liver disease: NAFLD; ochratoxin A: OTA; gene ontology: GO; Kyoto Encyclopedia of Genes and Genomes: KEGG; protein–protein interaction: PPI; albumin: ALB; glyceraldehyde-3-phosphate dehydrogenase: GAPDH; interleukin 6: IL6; tumor necrosis factor: TNF; actin beta: ACTB; estrogen receptor 1: ESR1; advanced glycation end-products-receptor for advanced glycation end-products: AGE-RAGE; phosphatidylinositol 3-kinase–protein kinase B: PI3K-Akt; interleukin-17: IL-17; metabolic dysfunction-associated steatotic liver disease: MASLD; International Agency for Research on Cancer: IARC; nuclear factor erythroid 2-related factor 2: Nrf2; antioxidant response element: ARE; nuclear factor kappa-light-chain-enhancer of activated B cells: NF-κB; tumor necrosis factor-α: TNF-α; interleukin-1β: IL-1β; insulin receptor substrate-1: IRS-1; traditional Chinese medicine: TCM; Comparative Toxicogenomics Database: CTD; adverse outcome pathways: AOPs; LAC: local average connectivity; Generally Recognized as Safe: GRAS; Food and Drug Administration: FDA; Acceptable Daily Intake: ADI; lipopolysaccharide: LPS; poly(lactic-co-glycolic acid): PLGA.
